# Efficiency of whole genome amplification of single circulating tumor cells enriched by CellSearch and sorted by FACS

**DOI:** 10.1186/gm510

**Published:** 2013-11-29

**Authors:** Joost F Swennenhuis, Joke Reumers, Kim Thys, Jeroen Aerssens, Leon WMM Terstappen

**Affiliations:** 1 Department of Medical Cell BioPhysics, MIRA Institute, University of Twente, Carre Room C4437, Hallenweg 23, 7522 NH Enschede, The Netherlands; 2 Janssen Research & Development, Janssen Pharmaceutical Companies of Johnson & Johnson, Beerse, Belgium

## Abstract

**Background:**

Tumor cells in the blood of patients with metastatic carcinomas are associated with poor survival. Knowledge of the cells’ genetic make-up can help to guide targeted therapy. We evaluated the efficiency and quality of isolation and amplification of DNA from single circulating tumor cells (CTC).

**Methods:**

The efficiency of the procedure was determined by spiking blood with SKBR-3 cells, enrichment with the CellSearch system, followed by single cell sorting by fluorescence-activated cell sorting (FACS) and whole genome amplification. A selection of single cell DNA from fixed and unfixed SKBR-3 cells was exome sequenced and the DNA quality analyzed. Single CTC from patients with lung cancer were used to demonstrate the potential of single CTC molecular characterization.

**Results:**

The overall efficiency of the procedure from spiked cell to amplified DNA was approximately 20%. Losses attributed to the CellSearch system were around 20%, transfer to FACS around 25%, sorting around 5% and DNA amplification around 25%. Exome sequencing revealed that the quality of the DNA was affected by the fixation of the cells, amplification, and the low starting quantity of DNA. A single fixed cell had an average coverage at 20× depth of 30% when sequencing to an average of 40× depth, whereas a single unfixed cell had 45% coverage. GenomiPhi-amplified genomic DNA had a coverage of 72% versus a coverage of 87% of genomic DNA. Twenty-one percent of the CTC from patients with lung cancer identified by the CellSearch system could be isolated individually and amplified.

**Conclusions:**

CTC enriched by the CellSearch system were sorted by FACS, and DNA retrieved and amplified with an overall efficiency of 20%. Analysis of the sequencing data showed that this DNA could be used for variant calling, but not for quantitative measurements such as copy number detection. Close to 55% of the exome of single SKBR-3 cells were successfully sequenced to 20× depth making it possible to call 72% of the variants. The overall coverage was reduced to 30% at 20× depth, making it possible to call 56% of the variants in CellSave-fixed cells.

## Background

Treatment options for patients with metastatic carcinomas are increasing rapidly and create a concomitant need for companion diagnostics to establish the therapy that is most likely to be effective. For a targeted therapy to be effective, its target needs to be present in the tumor cells. However, cancer cells are heterogeneous both within and between patients, forcing the need for individual characterization of the tumor cells. Moreover, during the course of the disease, resistance to therapy can develop and a timely detection and search for alternative therapies is desirable. Tumor biopsies are difficult if not impossible to obtain at the time a new line of therapy is indicated. Tumor cells from solid tumors are shed into the circulation and these circulating tumor cells (CTC) may serve as a liquid biopsy to guide therapy. The presence of CTC in patients with metastatic carcinomas is associated with poor survival, with a greater load indicating a worse prognosis [1-5]. Treatment targets can be assessed on CTC [6-9]; however, the frequency of CTC is extremely low [10,11] making it challenging to obtain a sufficient number of CTC to evaluate all potential treatment targets. The ability to isolate and amplify DNA from the individual CTC would overcome some of these challenges. We evaluated the feasibility of DNA amplification after fluorescence-activated cell sorting (FACS) of CTC obtained by what is currently the only clinically validated system for CTC enumeration [12].

## Methods

### Patient and control samples

The patient samples came from 10 patients with metastatic small cell lung cancer or metastatic non-small-cell lung cancer. The control samples were taken from healthy volunteers aged 20 to 55 years. From each participant, 10 ml of blood was drawn in a CellSave (Veridex LLC, Raritan, NJ, USA) or ethylenediaminetetraacetic acid (EDTA; Beckton Dickinson, Franklin Lanes, NJ, USA) evacuated blood draw tube. The healthy volunteers provided informed consent prior to donating blood under a study protocol approved by the Ethics Committee (METC Twente). All patients consented to provide blood for the study, and the study protocol was approved by the ethics review committee from University Medical Center Groningen, The Netherlands.

### Circulating tumor cell identification and preparation for cell sorting

Aliquots of 7.5 ml of blood were processed on a CellTracks Autoprep using the CellSearch Circulating Tumor cell kit (Veridex LLC) [12]. The enriched cells were fluorescently labeled with the nucleic acid dye 4ʹ 6-diamidino-2-phenylindole (DAPI) and the monoclonal antibodies directed against CD45 fluorescently labeled with allophycocyanin (APC) and directed against cytokeratins (CKs) labeled with phycoerythrin. For CTC enumeration, the cartridges were placed on a CellTracks Analyzer II or CellSpotter for image acquisition and image review (Veridex LLC) [11,12]. After scanning, the cartridges were stored at 4°C for a maximum of 24 hours before further processing. The content of the cartridge was transferred to a 12 × 35 mm flowtube and washed twice with 200 μl dilution buffer to ensure removal of the majority of cells from the cartridge. To ensure sufficient fluorescent nucleic acid signals, 2 μg/ml Hoechst 33342 (Molecular Probes, Eugene, OR, USA; cat. H3570) was added for 15 minutes at 37°C prior to cell sorting. In addition, 2 × 10^4^ beads (BD Biosciences, Jan Jose, CA, USA; cat. 345249) were added to ensure correct instrument settings and to serve as negative controls for cell sorting and DNA amplification.

### Single cell sorting

A FACSARIA II (BD Biosciences) equipped with a 375 nm, 488 nm and 633 nm laser and single cell deposit unit was used for single CTC sorting. Cells were sorted into 384 well plates (BioRad, Hercules, CA, USA; cat. HSP3805). The instrument was calibrated using CS&T beads (Beckton Dickinson; cat. 641412) before use. Before sorting, the sort gates and the number of cells and beads to be sorted into the 384 wells were set. For single cell sorting, 20 beads were also sorted into the well. To serve as positive and negative controls for DNA amplification, for each patient, 10 single leukocytes (CD45+, Hoechst+), five wells with 20 beads were sorted and five wells were left empty. Wells that had DNA amplification initiation within 95 minutes were regarded as positive.

### Efficiency of DNA amplification

Cells from the breast cancer cell line SKBR-3 were used to monitor the individual amplification reactions of both single and multiple cells compared to the amplification of background DNA. After trypsinization, the cells were suspended in 9 ml of culture medium (Dulbecco’s modified Eagle’s medium; Sigma Aldrich, St Louis, MO, US; cat. D5796), 1% penicillin/streptomycin (Sigma Aldrich; cat. P4333), 2 mM L-glutamine (Sigma Aldrich; cat. G7513) and 10% fetal calf serum (Sigma Aldrich; cat. F4135). This suspension was transferred to a CellSave tube. After 24 hours, 1 ml of this suspension was stained with 2 μg/ml Hoechst 33342 (Invitrogen, Grand Island, NY, USA; cat. H3570) for 15 minutes at 37°C. Next, 2 × 10^4^ beads (BD; cat. 345249) were added to the cells and 100, 10, 1 and 0 SKBR-3 cells and 20 beads were sorted into a 384-well plate. Five experiments were performed for each spike level except for the single cells, for which 10 were performed.

After sorting, the wells were treated with proteinase K by incubating with 1 μl 0.625% proteinase K (Sigma Aldrich; cat. P4850) in 10 mM Tris-HCl pH 7.4 for 1 hour at 50°C. The proteinase K was inactivated by incubating for 10 minutes at 96°C. After this step, 5 μl of an amplification mix containing Evagreen, a double-stranded DNA dye (Biotium, Hayward, CA, USA; cat. 31000), was added to each well. The amplification mix consisted of components from the GE Illustra GenomiPhi DNA amplification kit (GE Healthcare Life Sciences, Waukesha, WI, USA; cat. 25-6600-31) combined with Evagreen to monitor the reaction. The composition of the mix for each well was 1.75 μl sample buffer, 2.25 μl reaction buffer, 0.25 μl enzyme, 0.125 μl Evagreen, and 0.625 μl H_2_O. The plate was incubated for 250 minutes at 30°C in a BioRad CFX 384 quantitative PCR instrument while measuring the fluorescence every 5 minutes. After this, the Phi29 enzyme was inactivated for 10 minutes at 65°C and the plates stored at -20°C.

### Sample quality analysis of fixed cells, unfixed cells and isolated DNA by exome sequencing

SKBR-3 cells were trypsinized, suspended in culture medium and the genomic DNA isolated using the Promega Wizard SV Genomic DNA Purification System (Promega, Madison, WI, USA); cat. A1120). The cells were stained with Hoechst 33342 and individually placed in the wells of a 30-well epoxy-coated microscope slide (Menzel-Gläser, Braunschweig, Germany; cat X1XER312B) using a micromanipulator (Eppendorf, Hamburg, Germany). Confirmation that the wells indeed contained a single cell was obtained by fluorescence microscopy. Control wells were filled with 50 CellSave-fixed or 50 unfixed cells. Fixed cells were lysed by incubating with 1 μl 0.625% proteinase K in 10 mM Tris-HCl pH 7.4 for 1 minute, 10 minutes, 30 minutes or 1 hour at 50°C. The proteinase K was inactivated as above, by incubating for 10 minutes at 96°C. The unfixed cells were lysed by adding 1 μl lysis buffer (400 mM KOH, 25 mM EDTA, 100 mM dithiotreitol) to the cells and incubating for 10 minutes on ice, after which 1 μl neutralization buffer (0.4 μl 1 M HCl and 0.6 μl 1 M Tris-HCl, pH 7.5, premixed) was added. DNA was amplified as described above except for the addition of Evagreen. Samples were incubated for 2 hours at 30°C in a humidified chamber. Separately, 10 ng of the genomic DNA was amplified in a tube in 5 μl amplification mixture. To obtain enough DNA for sequencing, the initial amplifications samples were transferred to a vial and reamplified in a 100 μl amplification mixture using the same kit and reagent composition for 2 hours at 30°C. The final product was purified using a Qiagen PCR Purification Kit (Qiagen GmbH, Hilden, Germany). Genomic DNA (×1), amplified genomic DNA (×1), amplified DNA of 50 cells (×3) and amplified DNA of single cells (×7) were used for Illumina sequencing (Genome Analyzer IIx (GAIIx); Illumina, (San Diego, CA, USA)).

### Exome sequencing

Exomes were captured using Illumina’s TruSeq Exome Enrichment Kit. The TruSeq capture regions encompassed 62 Mb, including 20,794 genes (201,121 exons). According to the RefGene definitions, 94.4% of exonic regions, 83.9% of 5′-untranslated regions and 91.9% of 3′-untranslated regions were included in the targeted capture. Pre-enrichment DNA libraries were constructed according to the standard protocol from Illumina’s TruSeq DNA Sample Preparation Guide. A 200- to 300-bp band was gel-selected for each library and exome enrichment was performed according to Illumina’s TruSeq Exome Enrichment Guide. Two rounds of biotinylated bait-based hybridizations were performed, followed by binding with streptavidin magnetic beads, a washing step, and an elution step. A 10-cycle PCR enrichment was performed after the second elution and the enriched libraries were subjected to Illumina sequencing (GAIIx). Libraries were denatured with sodium hydroxide and loaded onto an Illumina cBot for cluster generation according to the manufacturer’s recommended protocols. Paired-end sequencing (2 × 75 bp) was performed using TruSeq SBS kits (Illumina). A single lane was used for each sample. The Burrows-Wheeler aligner [13] was used to align the raw reads from each sequencing lane (in fastq format) to the human reference genome (NCBI37/hg19) using default parameters. Aligned reads were processed and sorted using SAMtools [14] and PCR duplicates were marked with Picard MarkDuplicates [15]. Base recalibration, local realignment around indels and single nucleotide variant calling were performed using the GenomeAnalysis ToolKit [16]. Basic statistics for coverage depth and variant composition were performed using the BEDtools suite [17], VCFtools [18] and custom Perl scripts.

The sequence data have been deposited in the European Nucleotide Archive with a study accession number of PRJEB4979.

## Results

### Single cell sorting

**Figure 1:**
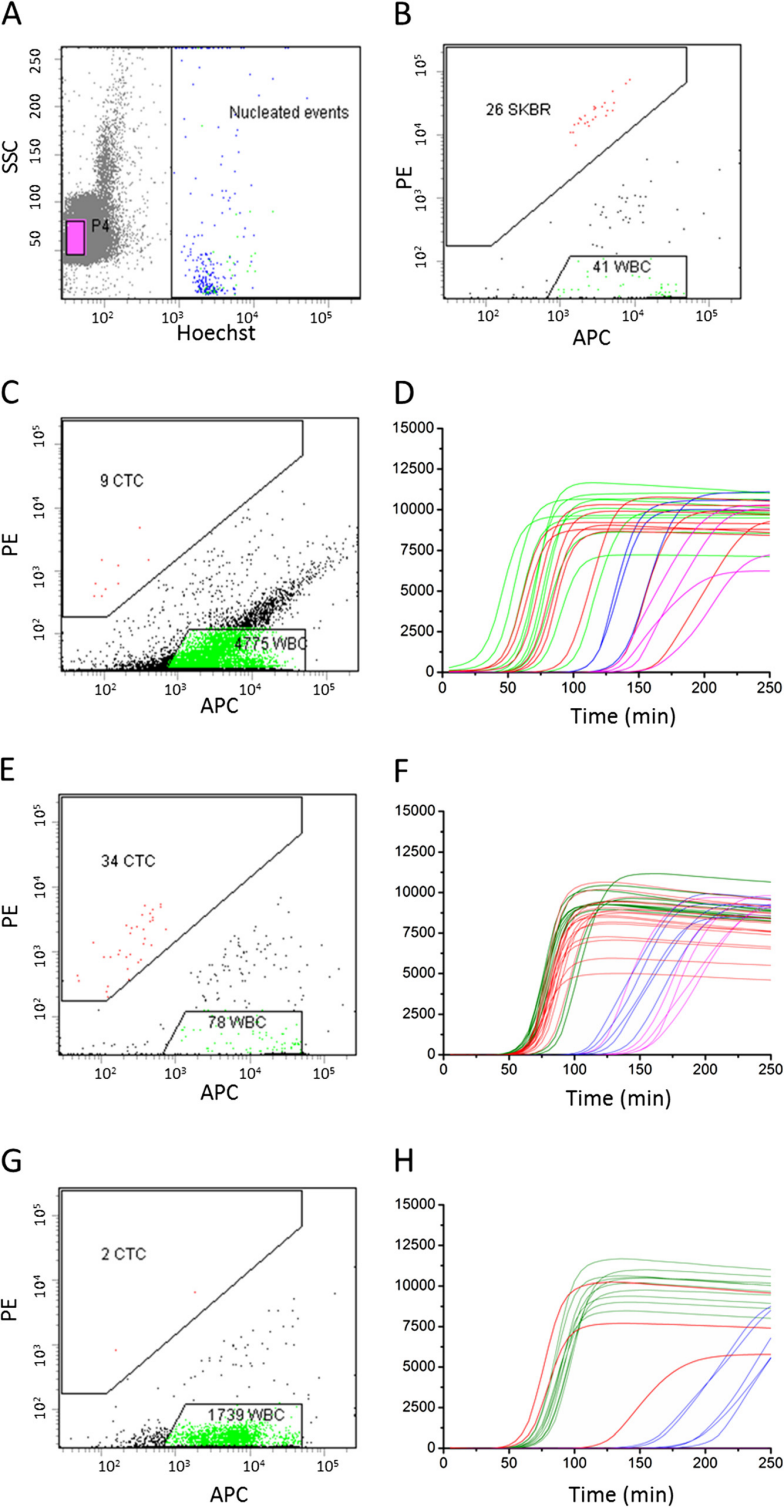
**Flow-cytometric analysis of CellSearch-enriched circulating tumor cells and real-time whole genome amplification of single-sorted leukocytes and circulating tumor cells.** Panels **(A)** and **(B)** show the sort gates to identify and sort single CTC, single leukocytes and beads. Panels **(C)** (Table 1, patient 7), **(E)** (Table 1, patient 1) and **(G)** (Table 1, patient 2) show the analysis and sort gates of three patients with lung cancer. Panels **(D)**, **(F)** and **(H)** show the corresponding real-time DNA amplification of the individual sorted cells. The curves show the Evagreen fluorescence of the whole genome amplification reaction mixes in time. APC = allophycocyanin, PE = phycoerythrin, SSC = sidescatter.

Figure 1 shows typical scatter plots from a flow-cytometric measurement of the contents of a CellSearch cartridge after processing 7.5 ml of blood spiked with 50 SKBR-3 cells. Panel A shows the DAPI/Hoechst staining and the gate to identify the beads is depicted in pink; the gate used to identify nucleated cells is also indicated. Panel B shows the CD45-APC staining versus the CK-PE staining of the DAPI/Hoechst + events. In this panel, the gates used to identify CTC (depicted in red) and leukocytes (depicted in green) are indicated. In this example, 41 events are identified as CD45+, CK- and DAPI + leukocytes, and 26 events as CD45-, CK + and DAPI + CTC. In this case, the CTC were the SKBR-3 cells that were spiked into the blood; 26 of the 50 (52%) spiked SKBR-3 cells were recovered. The shape and position of the CTC gate were set such that as few as possible DAPI + events appear in this gate when processing the blood of healthy donors while capturing as many CTC as possible from positive patient samples. Table S5 in Additional file 1 contains the results of eight healthy control samples analyzed with these settings after a CellSearch run. On average, these samples had 1.6 ±1.1 events in the CTC gate. In panels C, E and G, the CD45-APC, CK-PE scatterplots of samples from three patients with metastatic lung cancer are shown. A large variation in the number of CTC as well as the number of leukocytes in these samples can be observed. In addition, a population of CD45+, CK+, DAPI + events can be observed between the leukocytes and the CTC gates that vary in frequency.

### Efficiency of DNA amplification

**Figure 2:**
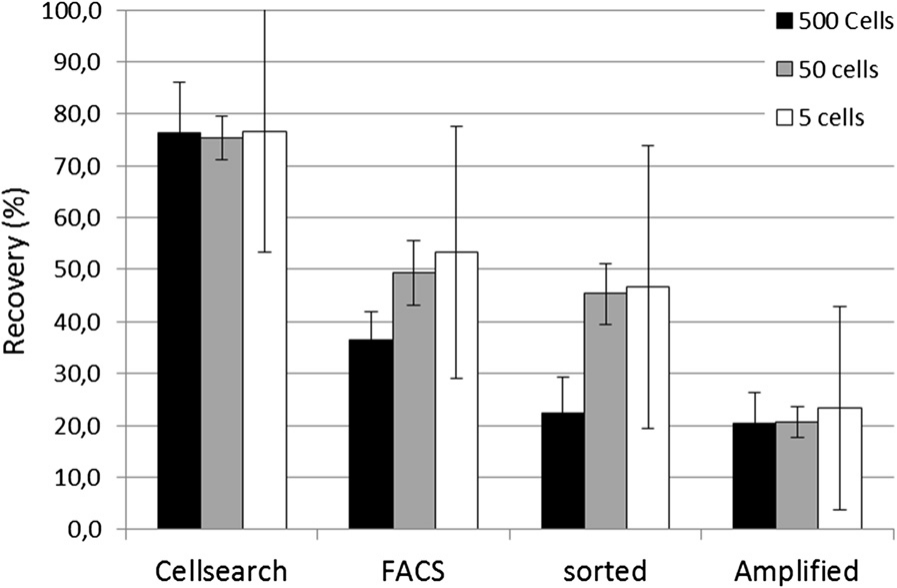
**DNA amplification SKBR-3 sorted into a 384-well plate.** Pink indicates 100 SKBR-3 cells plus 20 beads. Blue indicates 10 SKBR-3 cells plus 20 beads. Red indicates one SKBR-3 cell plus 20 beads. Green indicates 0 SKBR-3 cells and 20 beads. Black indicates an empty well. Cells were lysed by treating for 1 hour at 50°C with 1 μl protein K solution, 10 minutes inactivation at 96°C and cooled to 4°C. Whole genome amplification mix (5 μl) was added and incubated for 250 minutes at 30°C on a BioRad CFX 384 quantitative PCR machine while measuring fluorescence every 5 minutes. The two arrows point to the amplification of two single cells that did not show any amplification.

One of the main challenges in single-cell whole genome amplification (WGA) is quality control. Most amplification reactions are so sensitive that any trace of DNA in wells not containing a cell will be amplified to a maximum yield that is often indistinguishable from wells containing cells, even when using quality control methods such as gel electrophoresis or quantitative PCR. However, a simple method to check for the presence of a cell in a well is the addition of a fluorescent nucleic acid dye to the amplification reaction that can be followed in real time as shown in Figure 2. This figure shows the real-time monitoring of DNA amplifications in wells into which 100, 10, 1 and 0 SKBR-3 cells were sorted. Twenty beads were also sorted into each well. Besides acting as a control, the volume of the droplets serve as a carrier for the cell to move to the bottom of the well. The higher amount of DNA in wells with more cells can be clearly distinguished and, most importantly, the wells containing one cell can easily be distinguished from the negative controls. In two of the 10 wells (indicated by arrows) in which a single SKBR-3 cell was sorted, the amplification occurred at the same time as the negative controls, indicating that either the cell was not sorted or not lysed correctly. The negative controls all amplified at around 90 minutes regardless of whether they contained beads or not. One well with a single SKBR-3 did not show any amplification.

### Yield and reproducibility of circulating tumor cell isolation and whole genome amplification of spiked samples

**Figure 3:**
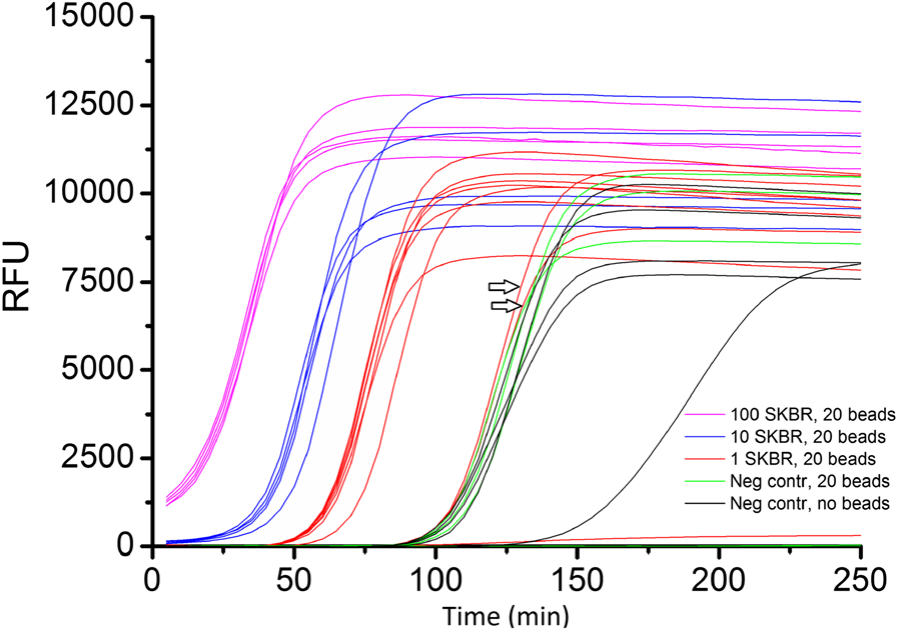
**Yields of each of the steps for whole genome amplification.** SKBR-3 cells were sorted into aliquots of 7.5 ml of blood containing 500, 50 and 5 cells, and enriched and enumerated by CellSearch. The contents of the CellSearch cartridge was placed in a FACS tube and SKBR-3 cells sorted into a 384-well plate. On each well, a GE GenomiPhi amplification reaction was performed in the presence of Evagreen. The yield of each step in the procedure was determined and plotted as a percentage of the starting amount. Each experiment was performed in triplicate except for the 5-cell sort, which was done six times. FACS, fluorescence-activated cell sorting.

Since CTC are rare in most patients, the goal is to keep the loss of cells during the procedure as low as possible. To quantify the yield and illustrate the reproducibility of each of the important steps, 500, 50 and 5 SKBR-3 cells were placed in 7.5 ml of blood. The blood was processed by the CellSearch system, transferred and identified by FACS, sorted by FACS, then DNA isolated and amplified by WGA. Figure 3 shows the yield of each of these steps. The efficiency of the CellSearch system to recover SKBR-3 cells was approximately 80% and close to the reported recovery of SKBR-3 cells [11,12]. Around 50% of the spiked SKBR-3 cells were then found back in the DAPI+, CK+, CD45- gate and about 40% of the spiked SKBR-3 cells were sorted. The yield at the 500 cell spikes was lower as compared to the 50 and 5 cell spikes. The percentage of wells that resulted in an efficient amplification by WGA was approximately 20% with the largest variation at the 5 cell spike level. More detailed information on the yield and reproducibility tests can be found in Table S1 in Additional file 1.

### Efficiency of circulating tumor cell isolation and whole genome amplification in patients with non-lung cancer

The number of CTC in patients with metastatic small cell and non-small-cell lung cancer varies greatly [1,2]. To determine the efficiency at which CTC can be isolated and individually sorted and the DNA amplified, blood samples from 10 patients with lung cancer were processed using the CellSearch system. The number of CTC identified in 7.5 ml of blood, the percentage of those cells that were identified as CTC by FACS and sorted, as well as the number of single CTC successfully amplified was determined (Table 1). An amplification was regarded as successful when a well had a cycle threshold (Ct) lower than the cutoff of 95 minutes. From the control samples that were done, two out of 50 negative controls with 20 beads sorted from the same samples were below this cutoff. None of the 50 empty negative controls was below this cutoff. Of the single leukocyte controls from the same patients, 85% (n = 100) were below this cutoff. Panels C, E and G in Figure 1 show three examples of the sort gates used and the real-time DNA amplification plots from these patients are shown in panels D, F and H. From the nine CTC identified by FACS in panel C, six showed a successful DNA amplification whereas nine of the ten leukocytes amplified successfully, as shown in panel D. From the 34 CTC identified by FACS in panel E, 17 showed a successful DNA amplification whereas all of the 10 leukocytes amplified successfully, as shown in panel F. From the two CTC identified by FACS in panel G, two showed a successful DNA amplification and also all of the ten leukocytes amplified successfully, as shown in panel H.

**Table 1:** CellSearch circulating tumor cell counts of samples from 10 patients with metastatic lung cancer, and the number of circulating tumor cells identified and sorted by fluorescence-activated cell sorting and successfully amplified

**Patient**	**Disease**	**CellSearch-**	**FACS-**	**FACS-**	**DNA-**	**DNA-**
		**identified**	**identified**	**sorted**	**amplified**	**amplified**
		**CTC**	**CTC**	**CTC**	**CTC**	**WBC (%)**
1	SCLC	81	34 (42%)	17 (21%)	17 (21%)	10 (100%)
2	SCLC	11	2 (18%)	3 (27%)	2 (18%)	10 (100%)
3	SCLC	3	3 (100%)	2 (67%)	2 (76%)	7 (70%)
4	NSCLC	4	2 (50%)	9 (225%)	0 (0%)	10 (100%)
5^a^	NSCLC	8	6 (75%)	32 (400%)	3 (38%)	2 (20%)
6	SCLC	37	6 (16%)	5 (14%)	4 (11%)	10 (100%)
7	SCLC	23	9 (39%)	9 (39%)	6 (26%)	9 (90%)
8	SCLC	41	17 (41%)	17 (41%)	16 (39%)	8 (80%)
9	SCLC	44	3 (6.8%)	2 (4.5%)	2 (4.5%)	9 (90%)
10	NSCLC	0	1	1	1	10 (100%)
Total		252	83 (33%)	97 (39%)	52 (21%)	85 (85%)

^a^The first five samples were used to optimize the gates for patient CTC sorting. After five samples, the gates were set and not changed anymore. This table shows the CTC identified after the gates were changed, showing the number of CTC that would have been sorted with these gate settings. The number of sorted cells could be higher than 100% because there were more cells in the gate due to the initial gate setting. For each patient, 10 leukocytes were also sorted to serve as a control. CTC, circulating tumor cells; FACS, fluorescence-activated cell sorting; NSCLC: non-small-cell lung cancer; SCLC: small cell lung cancer; WBC, white blood cells.

### Quality of the whole genome amplified DNA by exome sequencing

**Figure 4:**
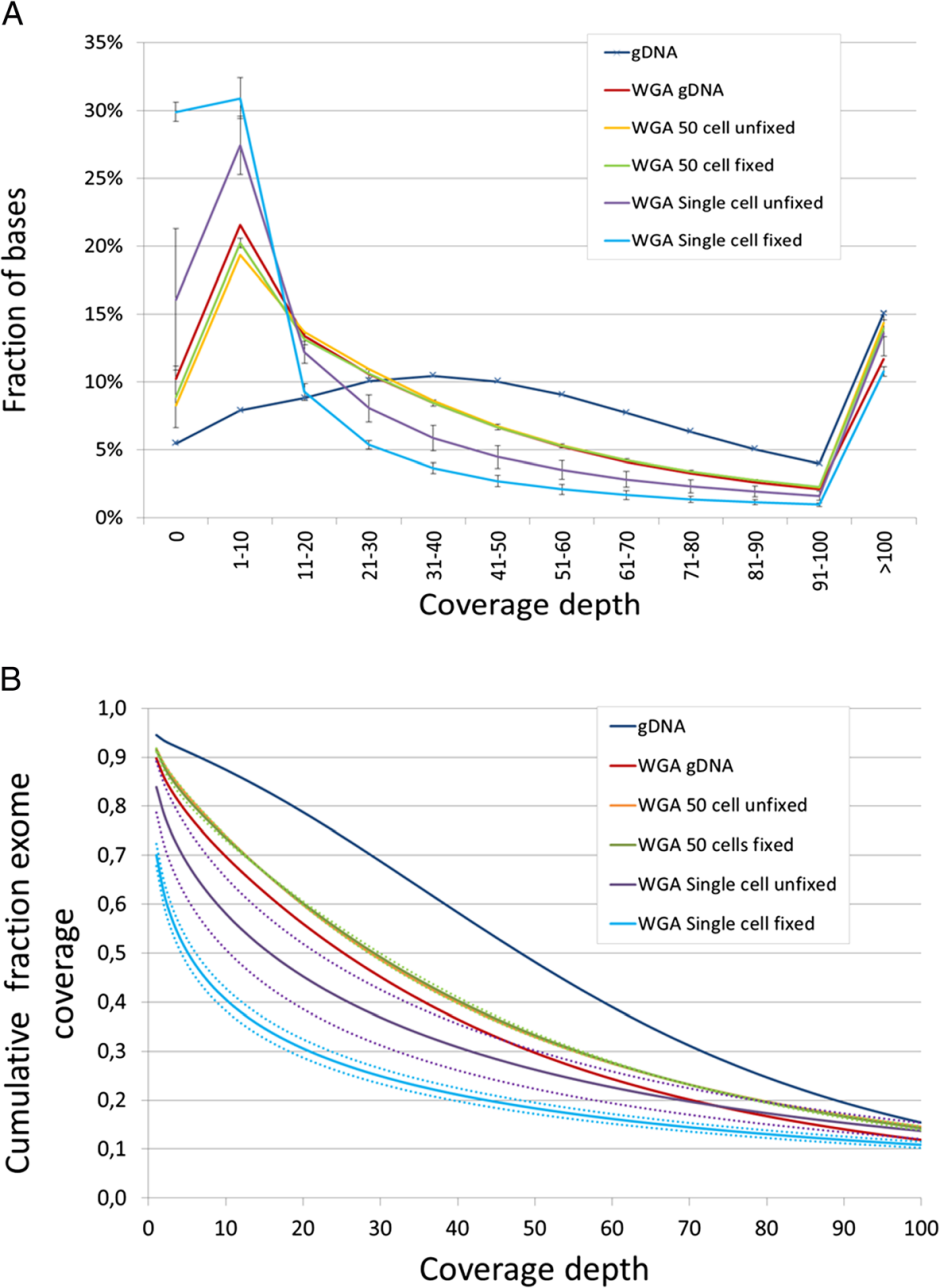
**Coverage depth. (A)** Fraction of bases with indicated coverage depth of genomic DNA (dark blue with mark, n = 1), amplified genomic DNA (red, n = 1), amplified DNA from multiple unfixed cells (yellow, n = 1), amplified DNA from multiple fixed cells (green, n = 2), and single unfixed (purple, n = 3) and fixed cells (blue, n = 4). **(B)** Cumulative fraction of the exome that was covered above coverage depths for the same samples as **(A)**. The dotted lines indicate the standard deviations. The lines from 50 cells fixed (green) and 50 cells unfixed (yellow) mostly overlap. gDNA, genomic DNA; WGA, whole genome amplification.

DNA produced by WGA kits is likely to contain aberrations introduced by the amplification method. The amplified genome of single cells is even more prone to aberrations because of the low starting copy number. DNA derived from single, multiple and genomic DNA from SKBR-3 cells was used for exome sequencing and analysis to check for the quality and representation of the exome. The fraction of bases mapping to the targeted exonic regions was fairly stable for all samples (approximately 60%) and the overall coverage depth per sample was sufficient for reliable variant calling. The coverage depth distribution (Figure 4A) shows that the fraction of uncovered bases at low coverage depth increased dramatically in all samples that had undergone WGA. For samples amplified from 50 cells there was no difference in coverage from fixed and unfixed cells. For samples amplified from single cells, there was a higher fraction of low or uncovered bases, highest for the DNA amplified from fixed single cells. Figure 4B shows the total coverage above specific coverage depths.

**Figure 5:**
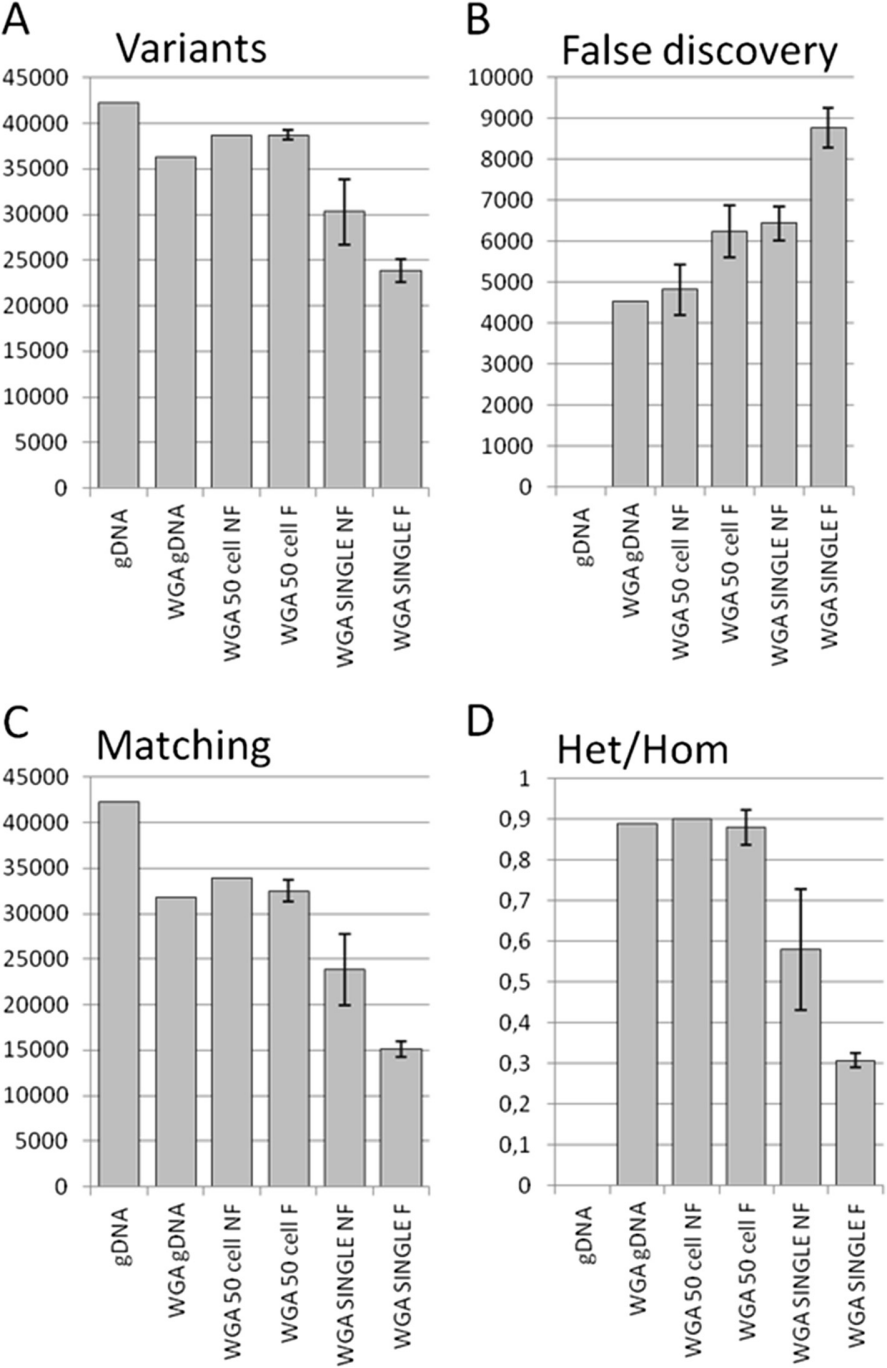
**Plots of the variants called in the DNA samples. (A)** Total number of variants found in the samples. **(B)** False discovery rate - the number of variants that were not in the genomic DNA. **(C)** The number of variants that match with the genomic DNA. **(D)** Heterogeneity to homogeneity ratio of the variants found. X-axis abbreviations: gDNA = genomic DNA, WGAgDNA = Whole genome amplification done on gDNA, WGA 50 cell whole genome amplification done on 50 cells, NF = not fixed, F = fixed.

In the genomic SKBR-3 DNA, 42,225 variants to the human reference genome (NCBI37/hg19) were found. In the WGA of this DNA, 36,339 variants were found, of which 75.3% matched the variants found in the isolated genomic DNA (Figure 5A,C). About the same number of variants (38,752) can be found in the DNA produced from the 50 cells, of which 78.1% matched the genomic DNA. In the single-cell samples, the number of matching variants decreased to 23,847 (56.5% matching) for unfixed cells and 15,071 (35.7% matching) for fixed cells. The false discovery rate was around 6,000 (range 4,534 to 6,438) for the multi-cell samples and the single-cell unfixed samples (Figure 5B). The single-cell fixed samples all had a clearly higher false discovery rate (range 8,267 to 9,211). In the single-cell samples, the ratio of heterozygous to homozygous variants also decreased (Figure 5D). More detailed information is provided on the variant calling and the false negatives in Tables S2 and S3 in Additional file 1.

## Discussion

To determine the type of cancer therapy with the greatest likelihood of success, tumor cells representing the cancer must be available at the time of therapy administration. Especially for targeted treatment, knowledge of the heterogeneity of tumor cells with respect to the treatment targets is of great importance. Characterization at the single-cell level is important to determine the extent of this heterogeneity. The ability to isolate tumor cells circulating in the blood presents an opportunity for a liquid biopsy throughout the course of the disease, and has spurred the development of a variety of technologies allowing such isolation. However, these CTC are extremely rare and the only clinically validated system to capture these cells is the CellSearch system. After CTC enrichment, 100 to 10,000 leukocytes remain among the CTC and various techniques can be used to isolate a single CTC from these enriched cell suspensions. Examples are the use of a micromanipulator [19] and the use of a DEPArray [20] after the cells have been removed from the cartridge and identified by fluorescence microscopy. Laser microdissection of single cells [21] is also an option, but no reported studies using this in combination with CellSearch exist to our knowledge. In our study, we evaluated the use of FACS to isolate single CTC from CTC-enriched cell suspensions. Each of these techniques has its advantages and disadvantages, and the choice is dictated by the availability of the equipment in the laboratory, the time and experience needed to isolate the single cells, and the costs and the efficiency at which CTC can be obtained from an enriched cell population.

All the techniques used to isolate CTC suffer from cell loss during the transfer steps. In our experiments, we lost 40% to 50% of the cells identified by the CellSearch system as CTC by FACS. This is still a favorable performance as compared to the cell losses we incurred using a micromanipulator (data not shown). The cell losses described are in a model system in which a known number of tumor cells are spiked in blood and the losses at each step can be determined. It is difficult, if not impossible, to determine losses when using patient blood because the number of CTC in the blood is not known. However, we can assess the number of CTC detected by the CellSearch system and determine the percentage of CTC recovered and the number of CTC from which the DNA can be successfully amplified. Successful amplification of the individually sorted CTC in samples from patients with lung cancer varied greatly, suggesting that the ‘quality’ of the CTC is an important factor. A likely cause of the variability is that the DNA cannot be successfully amplified when the CTC are undergoing apoptosis. A similar observation was made for CTC that were examined by fluorescence *in situ* hybridization [8]. Additional markers will need to be developed that can ensure the identified CTC have a high likelihood of achieving successful DNA/RNA isolation and amplification.

The processing time to obtain amplified DNA from single cells in general is around 48 hours. Since CTC are rare and in some cases fragile, EDTA blood is not optimal for conservation of such cells. For this reason, the CellSave tube was developed to keep the CTC stable for up to 96 hours before processing. The disadvantage of this fixation method is that DNA needs to be released from the fixed proteins of the cell for optimal amplification of the generated products. We used various incubation times for proteinase K treatment to optimize the sample pretreatment, measured in the quality of single-cell WGA DNA from cells exposed to CellSave by exome sequencing. However, this did not lead to any difference in the DNA sequence data. From earlier experiments, we know that treatment with proteinase K is necessary to achieve any amplification product from the single fixed cells. From this, we conclude that any further proteinase K treatment will not lead to further improvements.

It is desirable to characterize the DNA content of individual cells’ amplification of the genome without loss of representation of the original DNA. A variety of kits for DNA amplification are commercially available and can be divided into isothermal amplification kits (the Phi-29-based GE GenomiPhi kit or the Qiagen RepliG kit) and linker adapter based kits such as the Silicon Biosystems Ampli1 and the Rubicon PicoPLEX series. The first two have the advantage of a highly user-friendly protocol with very short hands-on time and low costs. The Rubicon PicoPLEX kit takes more effort, but is as fast as the isothermal kits. The Ampli1 kit has the longest hands-on time and has a protocol that stretches over three days. In fact, all the possible amplification techniques could be applied on the single-cell sorted CellSearch CTC. To demonstrate the sorting, amplification and sequencing of the single CTC, we adapted the GE GenomiPhi kit.

The analyzed exomes of the single SKBR-3 cells were adversely affected by the amplification process but still contained useful information. If we look at the sharp increase in low or uncovered bases after WGA in all samples, it is clear that the amplification process itself causes a large bias. This makes this amplified DNA unsuitable for quantitative measurements. Furthermore, the starting amount of one cell and the cell fixation also have a substantial influence on the final DNA quality. A part of the variants could be recovered and this could be increased when combining the DNA of individual amplified cells.

From Figure 3 it is clear that, within the same samples, there was a large difference in the number of detected cells in CellSearch and the subsequent number of CTC detected using FACS. It could be that the cells were lost due to their fragility, in turn due to the permeabilization process in the CellSearch protocol or by nonspecific sticking of the cells to surfaces of pipettes and the tube. Furthermore, CTC that are connected to a leukocyte might miss the CTC gate due to the level of CD45 APC staining being too high. We learned from earlier studies that a variable number of CTC in patients are apoptotic [8] and the effect of this process on the genomic sequence needs to be investigated. The amplification method of choice described in this paper is just one of many available methods. The method used depends on the sample type and quality but also the type of output data that are required. The differences between these kits and the most optimal kit for single-cell CTC DNA amplification are yet to be determined.

## Conclusions

We have shown that CTC enriched by the CellSearch system can be single-cell-sorted by FACS, and the DNA retrieved and amplified with an overall efficiency of 20%. From patient samples that were positive for CTC, it was possible to sort and amplify 20.6% of the CTC found in CellSearch. Analysis of sequencing data shows that GenomiPhi-amplified DNA can be used for variant calling, but not for quantitative measurements. Close to 45% of the exome of single cells can be successfully sequenced to 20× depth, making it possible to call 72% of the variants. Overall, coverage was reduced to 30% at 20× depth making it possible to call 56% of the variants in CellSave-fixed tumor cells.

## Abbreviations

APC: Allophycocyanin; bp: Base pairs; CK: Cytokeratin; CTC: Circulating tumor cells; DAPI: 4'-6-diamidino-2-phenylindole; EDTA: Ethylenediaminetetraacetic acid; FACS: Fluorescence activated cell sorting; Mb: Megabases; PCR: Polymerase chain reaction; PE: Phycoerythrin; WGA: Whole genome amplification.

## Competing interests

JR, KT and JA are employees of Janssen Pharmaceutical Companies of Johnson & Johnson. Veridex LLC is also a Johnson & Johnson company. LT is a consultant for Veridex LLC, which markets and sells the CellSearch system used in this study. JS is a member of the MCBP group. The MCBP group of the University of Twente receives research funding from Veridex LLC. The authors declare that they have no other competing interests.

## Authors’ contributions

JS carried out the spiking, isolation and DNA amplification experiments and drafted the manuscript. JR and KT were responsible for the sequencing and the sequence data analysis and approved the final manuscript. JA was responsible for the design of the experiments needed for sequencing and approved the final manuscript. LT was responsible for the overall study design and drafting of the manuscript. All authors had full access to all the data and the corresponding author had final responsibility for the decision to submit for publication. All authors read and approved the final manuscript.

## Supplementary Material

Additional file 1: Table S1 Detailed data of the efficiency testing of FACS sorting and whole genome amplification success rate. **Table S2**: Detailed data on the variant calling of all the samples. **Table S3**: False negative variants in the 50- and single-cell samples. **Table S4**: False negative variants in the reference sample. **Table S5**: FACS data negative healthy control samples. Click here for file
