# Supplementary material for: Efficiency of whole genome amplification of single circulating tumor cells enriched by CellSearch and sorted by FACS
Source: Genome Med. 2013 Nov 29;5(11):106. doi: 10.1186/gm510 (PMC3978840; doi:10.1186/gm510)
Supplement: Additional file 1: Table S1 — Detailed data of the efficiency testing of FACS sorting and whole genome amplification success rate. Table S2: Detailed data on the variant calling of all the samples. Table S3: False negative variants in the 50- and single-cell samples. Table S4: False negative variants in the reference sample. Table S5: FACS data negative healthy control samples. [file gm510-S1.docx]

Additional file

**Table S1 efficiency data**

| **Cartridge** | **Start** | **CellSearch** | | **FACS Detected** | | **Single cell sorted** | | | | **Amplified Total** | | |
| --- | --- | --- | --- | --- | --- | --- | --- | --- | --- | --- | --- | --- |
|  |  | **(%step) %total** | | **(%step) %total** | | **(%step) %total** | | | | **(%step) %total** | | |
| **01030300** | 500 | 404(80.8%) | 80.8% | 203(50.2%) | 40.6% | 86 (42.4%) | | 17.2% | | 30/30(100%) | | 17.2% |
| **01030301** | 500 | 327(65.4%) | 65.4% | 152(46.5%) | 30.4% | 100(65.6%) | | 20.0% | | 25/30(83.3%) | | 16.7% |
| **01030302** | 500 | 416(83.2%) | 83.2% | 192(46.1%) | 38.4% | 151(78.6%) | | 30.2% | | 27/30(90.0%) | | 27.2% |
| **Average** | 500 | 382(76.5%) | 76.5% | 182(47.6%) | 36.5% | 112(61.5%) | | 22.5% | | 27/30(90.0%) | | 20.4% |
| **Cartridge** | **Start** | **CellSearch** | | **FACS Detected** | | | **Single cell sorted** | | | | **Amplified** | |
| **01046645** | 50 | 36(72.0%) | 72.0% | 22 (61.1%) | 48% | | 21(95.4%) | | 42% | | 9 (42.8%) | 18.0% |
| **01046646** | 50 | 37(74.0%) | 74.0% | 24 (64.9%) | 48% | | 21(87.5%) | | 42% | | 12(57.1%) | 24.0% |
| **01046647** | 50 | 40(80.0%) | 80.0% | 28 (70.0%) | 56% | | 26(92.9%) | | 52% | | 10 (34.6%) | 18.0% |
| **Average** | 50 | 37.7(75.3%) | 75.3% | 25.3(67.1%) | 50.7% | | 22.7(30.2%) | | 45% | | 10.3(20.6%) | 20% |
| **Cartridge** | **Start** | **CellSearch** | | **FACS Detected** | | | **Single cell sorted** | | | | **Amplified** | |
| **01046569** | 5 | 5(100%) | 100% | 4(80%) | 80% | | 4(100%) | | 80% | | 0(0%) | 0% |
| **01046570** | 5 | 3(60%) | 60% | 3(100%)* | 100%* | | 2(66%) | | 40% | | 2(100%) | 40% |
| **01046572** | 5 | 4(80%) | 80% | 4(80%) | 100% | | 4(100%) | | 80% | | 2(50%) | 40% |
| **01046573** | 5 | 4(80%) | 80% | 2(50%) | 40% | | 1(50%) | | 20% | | 1(100%) | 20% |
| **01046575** | 5 | 5(100%) | 100% | 2(40%) | 40% | | 2(100%) | | 40% | | 2(100%) | 40% |
| **01046576** | 5 | 2(40%) | 40% | 1(50%) | 20% | | 1(100%) | | 20% | | 0(0%) | 0% |
| **Average** | 5 | 3.8(76.7%) | 76.7% | 2.6(53.3%) | 63.3% | | 2.3(86%) | | 47% | | 1.2(23.3%) | 20% |

*2 events were sorted which were just inside the gate but are probably no SKBR-3 cells judged by the lower cytokeratin level.

**Table S2: Variant calling data**

| **Sample** | **Fixation** | **Variants** | **Matching sites** | | | | **False discovery rate** | |
| --- | --- | --- | --- | --- | --- | --- | --- | --- |
| REF | Unfixed | 42225 | Total | % Matching | Homozygous | Heterozygous | FD | wrt exome |
| WGA | Unfixed | 36339 | 31805 | 75,32% | 16818 | 14978 | 4534 | 0,007% |
| 50CELL_02 | Unfixed | 38749 | 33929 | 80,35% | 17847 | 16074 | 4820 | 0,008% |
| 50CELL_03 | Fixed | 39146 | 33335 | 78,95% | 17401 | 15925 | 5811 | 0,009% |
| 50CELL_04 | Fixed | 38362 | 31684 | 75,04% | 17160 | 14516 | 6678 | 0,011% |
| SINGLE_01 | Unfixed | 32505 | 26799 | 63,47% | 15617 | 11176 | 5706 | 0,009% |
| SINGLE_02 | Unfixed | 26210 | 19345 | 45,81% | 13221 | 6122 | 6865 | 0,011% |
| SINGLE_03 | Unfixed | 32142 | 25398 | 60,15% | 16411 | 8982 | 6744 | 0,011% |
| SINGLE_04 | Fixed | 24920 | 15986 | 37,86% | 12112 | 3872 | 8934 | 0,014% |
| SINGLE_05 | Fixed | 24835 | 15624 | 37,00% | 11931 | 3692 | 9211 | 0,015% |
| SINGLE_06 | Fixed | 23031 | 14378 | 34,05% | 10979 | 3395 | 8653 | 0,014% |
| SINGLE_07 | Fixed | 22562 | 14295 | 33,85% | 11101 | 3191 | 8267 | 0,013% |

**Table S3: variant calling, false negatives**

| **Sample** | **Fixation** | **Variants** | **Matching sites** | | **False negatives** | |
| --- | --- | --- | --- | --- | --- | --- |
| REF | Unfixed | 42225 | Total | % Matching | (FN) | %FN |
| WGA | Unfixed | 36339 | 31805 | 75,32% | 6943 | 16,44% |
| 50CELL_02 | Unfixed | 38749 | 33929 | 80,35% | 4433 | 10,50% |
| 50CELL_03 | Fixed | 39146 | 33335 | 78,95% | 3874 | 9,17% |
| 50CELL_04 | Fixed | 38362 | 31684 | 75,04% | 5139 | 12,17% |
| SINGLE_01 | Unfixed | 32505 | 26799 | 63,47% | 12174 | 28,83% |
| SINGLE_02 | Unfixed | 26210 | 19345 | 45,81% | 20123 | 47,66% |
| SINGLE_03 | Unfixed | 32142 | 25398 | 60,15% | 13780 | 32,63% |
| SINGLE_04 | Fixed | 24920 | 15986 | 37,86% | 21994 | 52,09% |
| SINGLE_05 | Fixed | 24835 | 15624 | 37,00% | 22160 | 52,48% |
| SINGLE_06 | Fixed | 23031 | 14378 | 34,05% | 23641 | 55,99% |
| SINGLE_07 | Fixed | 22562 | 14295 | 33,85% | 24155 | 57,21% |

**Table S4: False negatives in the reference sample.** Variants not detected in the reference sample but consistenly detected in the 50- or single-cell samples may represent false negatives in the reference samples. Variants present in different sample groups are listed below and for each set of detected variants an estimate for the false negative rate (FNR) in the reference sample is given.

| **Variants not present in reference sample** | **Number of variants** | **FNR(%)** |
| --- | --- | --- |
| 50-cell samples (n=4) | 905 | 2.1% |
| 50-cell not fixed samples (n=2) | 1740 | 4.0% |
| 50-cell fixed samples (n=2) | 1663 | 3.8% |
| Single-cell samples (n=7) | 15 | 0.0% |
| Single-cell not fixed samples (n=3) | 407 | 1.0% |
| Single-cell fixed samples (n=4) | 60 | 0.1% |

**Table S5: FACS data negative healthy control samples.** Events detected in the CTC and the WBC gates from CellSearch prepped healthy control samples. Column # CTC CellSearch sums the number of CTC found in the CellSearch test. The Unassigned events are the number events selected by CellSearch but were no CTC. The CTC and WBC gated events are the events gated in the FACS.

| **Patient #** | **# CTC CellSearch** | **Unassigned events CellSearch** | **CTC Gated** | **WBC Gated** |
| --- | --- | --- | --- | --- |
| 1 | 0 | 18 | 0 | 1496 |
| 2 | 0 | 225 | 2 | 14953 |
| 3 | 0 | 19 | 1 | 332 |
| 4 | 0 | 30 | 3 | 295 |
| 5 | 0 | 50 | 1 | 311 |
| 6 | 0 | 5 | 3 | 485 |
| 7 | 0 | 16 | 1 | 279 |
| 8 | 0 | 28 | 2 | 580 |
| average | 0 | 49.1 | 1.6 | 2341 |
